# Evaluation of the “15 Minute Challenge”: A Workplace Health and Wellbeing Program

**DOI:** 10.3390/healthcare12131255

**Published:** 2024-06-24

**Authors:** Ben Singh, Ty Ferguson, Artem Deev, Anton Deev, Carol A. Maher

**Affiliations:** 1 Allied Health and Human Performance, University of South Australia, Adelaide, SA 5001, Australia; ty.ferguson@unisa.edu.au (T.F.); carol.maher@unisa.edu.au (C.A.M.); 2 15 Minute Challenge, Adelaide, SA 5000, Australia; artem.deev@15minchallenge.com.au (A.D.); anton.deev@15minchallenge.com.au (A.D.)

**Keywords:** mHealth, ehealth, physical activity, exercise, workplace, lifestyle, gamification

## Abstract

The 15 Minute Challenge is an mHealth workplace wellness initiative, employing gamification to promote physical activity, aiming to enhance health outcomes and overall well-being. This retrospective cohort study evaluated the effectiveness of the program among employees at various Australian, New Zealand, and UK workplaces. Real-world data from 11,575 participants across 73 companies were analyzed. The program encouraged daily 15 min physical activity sessions over six weeks. Participants self-reported their physical activity and fitness, energy, overall health, sleep quality, and mood at baseline and 6 weeks. Program satisfaction, engagement rates, and adherence to the program were also assessed. Effectiveness was evaluated using multi-level mixed-effects linear regression analyses. The intervention showed significant increases in physical activity, with 95% of participants meeting or exceeding international physical activity guidelines, up from 57% at baseline (*p* < 0.05). Self-reported fitness, energy, overall health, sleep quality, and mood significantly improved (between 7.1 and 14.0% improvement; all *p* < 0.05). High satisfaction was reported, with 92% of participants recommending the program. The 15 Minute Challenge effectively increased physical activity levels and improved self-reported health outcomes among participating employees. The high satisfaction rates and significant health improvements highlight the potential of workplace wellness programs to combat sedentary behavior and promote a healthier, more active lifestyle.

## 1. Introduction

Physical activity is a pivotal factor in preventing chronic diseases, reducing mortality risk, and increasing life expectancy [1]. Regular physical activity is crucial in preventing and managing various chronic conditions, including cardiovascular disease, type 2 diabetes, obesity, cancer, schizophrenia, and chronic obstructive pulmonary disease (COPD) [1,2,3]. The World Health Organization’s (WHO) 2020 guidelines recommend 150–300 min of moderate-intensity or 75–150 min of vigorous-intensity activity per week, which is associated with a 20–30% reduction in all-cause mortality risk [4]. Globally, about 23% of adults did not meet the 2016 WHO recommendation of 150 min of moderate-intensity or 75 min of vigorous-intensity activity per week [5]. The lack of physical activity is a global concern, recognized as the fourth leading risk factor for mortality worldwide [6,7]. WHO estimates indicate that insufficient physical activity causes about 3.2 million deaths and 32.1 million disability-adjusted life years (DALYs) annually, representing 2.1% of global DALYs. Stressing the importance of reducing sedentary behaviors, the guidelines note that any intensity of physical activity provides significant health benefits [8].

Implementing physical activity initiatives and policies within the workplace could provide extensive health benefits for employees and employers [9]. The modern workforce environment often involves prolonged sedentary time, which has been linked to higher risks of obesity, type 2 diabetes, heart disease, and other chronic conditions [10,11]. Simple strategies like providing activity breaks, walking meetings, or standing desks can get employees moving during work hours. Research shows that regular exercise at work helps reduce stress, boost energy levels, enhance mood and concentration, and decrease musculoskeletal discomfort that contributes to lost productivity [12]. Physically active employees take less sick time and have lower health care costs [13]. With a majority of adults spending much of their waking time at work, offices and workplaces represent ideal settings for promoting physical activity. Employers can enhance employee health and organizational performance and cost outcomes by providing exercise incentives, resources, and opportunities.

Workplace physical activity interventions have shown some evidence of effectiveness, although more research is needed to determine the most impactful components of these interventions [14,15,16]. Studies suggest that multi-strategy interventions, including information, education, activity, motivation, support, monitoring, and feedback, can increase physical activity in the workplace [14,15,16]. Workplace interventions such as financial incentives for physical activity [17] and pedometer-based walking programs [18] have been found to positively impact employees’ physical activity levels. Participation rates in previous workplace physical activity interventions have varied widely, ranging from 10% to 78% across different workplaces [19]. Therefore, strategies to maximize participation and engagement are an important consideration for future workplace interventions. Furthermore, previous workplace physical activity interventions have not fully utilized technology. Advances in wearables, mobile apps, and digital platforms provide new opportunities to incorporate these technologies to promote physical activity in the workplace.

The 15 Minute Challenge is a workplace wellness initiative that employs mHealth technology and behavioral economics to promote physical activity through social accountability [20]. Despite theoretical promise, the program’s effectiveness and implementation best practices have yet to be evaluated empirically. Rigorous comparative trials are necessary to validate impacts on objective health outcomes and sustainability. This initiative offers a unique approach to workplace physical activity promotion. This manuscript details a study assessing the 15 Minute Challenge program’s effectiveness in enhancing physical activity levels and health outcomes among employees. The program aims to foster regular physical activity through short, manageable exercise routines easily integrated into the workday. The 15 Minute Challenge shares some similarities with other workplace programs, such as promoting physical activity through self-organized teams and activity logging. However, it distinguishes itself by emphasizing a minimal daily commitment of just 15 min of activity, rather than focusing on step count or other metrics. This specific focus on micro-habits and ease of integration into daily routines aims to reduce barriers to participation and enhance long-term adherence. The program gamifies the experience through team competitions, activity logging, social sharing, and personal milestones within a mobile app. These elements leverage principles of behavioral economics and technology to motivate employees through social reinforcement, flexibility, and enjoyment [14,15,16].

The aim of this study was to conduct a retrospective analysis to evaluate the effectiveness of the 15 Minute Challenge for promoting increased physical activity and improving health outcomes among employees at various Australian, New Zealand, and UK workplaces. The primary objective of this study was to evaluate changes in employee physical activity participation and perceived health impacts following the 6-week intervention. Secondary objectives included determining variability in effectiveness across different corporations and occupations. It was hypothesized that the 15 Minute Challenge would lead to significant increases in physical activity levels and improvements in health outcomes among employees. A comprehensive analysis of the program’s effectiveness will contribute valuable insights to the growing evidence on workplace physical activity interventions and their potential to enhance employee health and well-being.

## 2. Materials and Methods

### 2.1. Design

A retrospective cohort design was used to assess the efficacy of a 6-week workplace wellness physical activity intervention implemented across various Australian, New Zealand, and UK workplaces. This research was deemed not to require ethical approval from the University of South Australia Institutional Review Board, as it was retrospective (Application ID: 205881). During enrollment in the intervention, participants provided consent for the utilization of their data for quality improvement purposes. The intervention is reported following the TIDieR Checklist (“Template for Intervention Description and Replication”) and the study is reported following the TREND statement (“Transparent Reporting of Evaluations with Nonrandomised Designs”).

### 2.2. Sample

This analysis involved 11,575 adults who were employees aged 18–65 years from 73 companies in Australia, New Zealand, and the UK which were offered the 15 Minute Challenge between February 2022 and September 2023. Corporations representing diverse industries including healthcare, financial services, higher education, technology, and others allowed solicitation of interested staff at the local campus/office level. Leadership at participating organizations agreed to internal promotion of the study opportunity through emails, staff meetings, posters, and existing wellness channels. The consenting participant sample consisted of employees from the workplaces that were invited to participate.

### 2.3. Intervention Overview

The 15 Minute Challenge is a 6-week online workplace physical activity competition encouraging participants to complete at least 15 min of exercise daily. Employees from workplace teams record activities via a mobile-responsive web platform.

The team-based structure, virtual accountability, and competition element aim to motivate increased activity through socially reinforced motivation. The 15 Minute Challenge represents a scalable, low-resource workplace wellness initiative leveraging principles of gamification, social accountability, and flexibility. Employees self-organize into workplace teams ranging from 3 to 8 people and access the responsively designed mobile/web app platform to log participation. Participants provide department/location information and baseline health measurements when registering for the program using the app.

**Figure 1:**
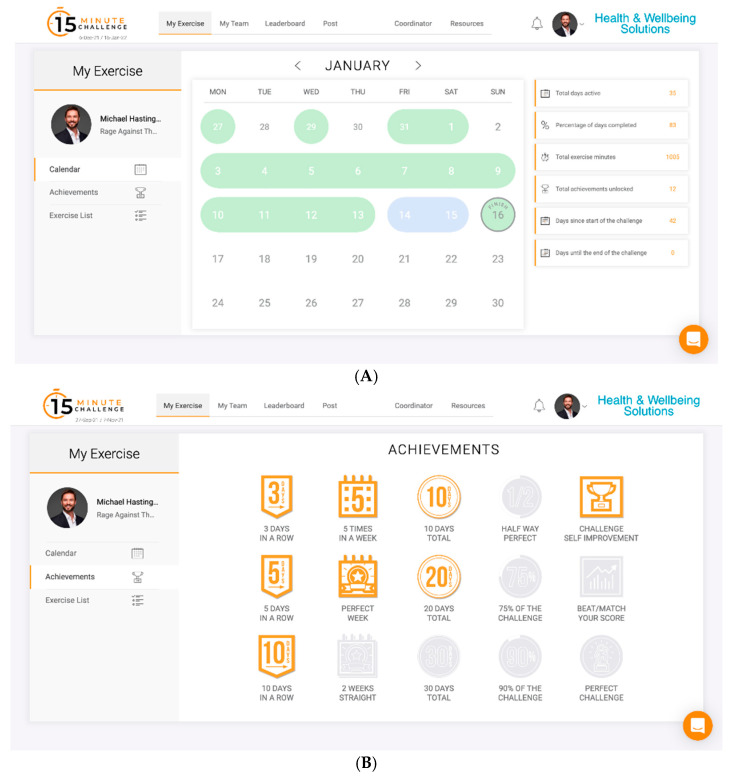
(**A**) My Exercise page—calendar for recording of exercises; (**B**) My Exercise page—individual achievement.

The app allows teams to track rankings, displaying cumulative participant exercise sessions for the organisation. Users log each completed bout of physical activity totalling ≥15 min in duration through selecting that day’s date, which updates the leader board (Figure 1A,B). Participants documented details including exercise type and duration. The app also provides an exercise idea library with illustrations and guidance to support activity planning and goal setting. The app utilized gamification principles like team competitions, daily activity logging, an exercise library with illustrations for activities such as walking, running, cycling, as well as specific resistance exercises (e.g., bodyweight squats, push-ups) and yoga, social sharing, and personal milestones to motivate employees to increase their physical activity levels through socially reinforced motivation, accountability, and flexibility.

A message board facilitates communication and photo sharing between teammates. Email reminders inform participants to log exercises, while personalized statistics detail individual and team performance analytics like streaks and accomplishments. The gamification elements like team rankings, daily logging, social features, and personal milestones aimed to motivate participants to increase their physical activity levels through friendly competition, social support, and goal setting. The app was used during both work and non-work hours by participants. Participants were encouraged to find time during work hours to be active (e.g., taking the stairs, going for a walk during their lunch break), as well as to be active at other times of the day when it suited them (out of work hours). Voluntary post-study surveys re-assess health metrics along with perspectives on experience.

The overarching intervention focuses on flexibility, allowing any form of cardiovascular activity based on personal preference to facilitate participation autonomy and enjoyment. By emphasizing consistency through manageable 15 min daily benchmarks rather than duration or intensity, the program aims to build sustainable activity habits. The team-based platform combined with structured reminders and reporting of participation provides the accountability framework to motivate regular engagement.

### 2.4. Measures

Health and wellbeing outcomes: Measurements were completed by participants at baseline and post-intervention using online self-report surveys that were built into the app. Single items asked participants to rate their perceived fitness, energy, mood, sleep quality, and overall health on a 10-point scale (higher score = more favorable outcome; see Table S1).

User feedback: User feedback post-intervention was gathered through additional satisfaction questions. These questions evaluated overall experience, perceived benefits, enjoyment, future interest, and potential for sustained impact, utilizing 5-point Likert agreements (strongly disagree, disagree, neutral, agree, strongly agree).

Usage data: Usage data were collected through various metrics, including frequency of interaction, time spent on the platform, and engagement with specific features. Analysis of usage patterns provided insights into user behavior and interaction with the intervention.

Engagement, retention, and satisfaction: Objective program analytics quantification included participation rate (proportion of enrolled individuals actively engaging in the program), retention rate (proportion of participants who completed the program), total exercise days logged (out of 42), average sessions per person, and percentage of participants that logged data in the final week. Nine questions evaluating participant satisfaction, perceived benefits, and willingness to continue or recommend the program were asked using a “Strongly Disagree” to “Strongly Agree” scale (See Table S1).

### 2.5. Statistical Analysis

Participant characteristics and physical activity data were analyzed descriptively using counts, percentages, medians, interquartile ranges (IQRs), and means. Self-reported pre- and during-program physical activity levels were categorized as either below, meeting, or exceeding international physical activity guidelines of 150 to 300 min of moderate-to-vigorous physical activity per week [4]. Where a full week of data was not available, per-day values were converted to a weekly total.

Data visualizations were created in MATLAB Version: 9.13.0 (R2022b, The MathWorks Inc, Natick, MA, USA) and Microsoft Excel (Version 2021) (Microsoft Corporation, Redmond, WA, USA). Baseline and end-of-program measures of health outcomes were compared using multi-level mixed-effects linear regression analyses. Analyses were completed in Stata 17 (StataCorp, College Station, TX, USA) with statistical significance set at 0.05. Random intercepts were used to account for the nested structure of the data (i.e., repeated measures within individuals and individuals within companies). Health outcomes were the dependent variables and were analyzed in separate models. Timepoint (i.e., baseline and end of program) was included as a fixed effect. The regression coefficients for health outcomes were used to identify the difference in self-reported health from baseline to the end of the program. To verify the internal consistency of the instrument across the analyzed dimensions, we calculated Cronbach’s alpha for each dimension as well as the overall score. Participant self-reported physical activity levels were collected pre-program and during the study period using surveys administered through the app. Chi-square tests were performed to assess changes in the proportion of participants meeting international physical activity guidelines from pre-program to during the program.

Program analytics quantified participation and retention rates, with comparison of pre-post self-reported health, wellbeing, and productivity indices. Participant evaluation was also conducted to assess program acceptability, including questions regarding program recommendation and overall satisfaction.

## 3. Results

### 3.1. Program Participation

Data were available for 73 companies who had participated in the program. These companies span a variety of industries (see Table S2 for additional details). Across all companies, there were 2105 registered teams (median per company = 19, IQR = 12–46) and 13,116 people expressed interest in participating in the program (median per company = 110, IQR = 63–248). Of the 13,116 who expressed interest, those who recorded at least one day of activity were eligible and included in this analysis.

### 3.2. Physical Activity

**Figure 2:**
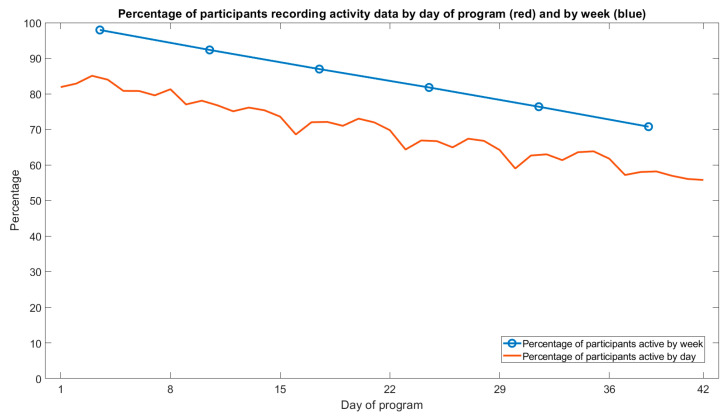
Percentage of participants recording activity data by day of program.

Across all companies, there were 11,575 participants (i.e., recording at least one day of activity; 88% of participants who expressed interest) who recorded 338,529 days of activity for a total of 19,169,511 min of physical activity. An average of 69.6% of participants recorded activity data each day, with a steady attrition across the program’s duration, ranging from 81.9% on day 1 to 55.8% on day 42 (see Figure 2, red line). In the first week of the program, activity data were recorded by 98% of participants, and 70.8% of participants logged activity data at least once in the final week (Figure 2, blue line).

**Figure 3:**
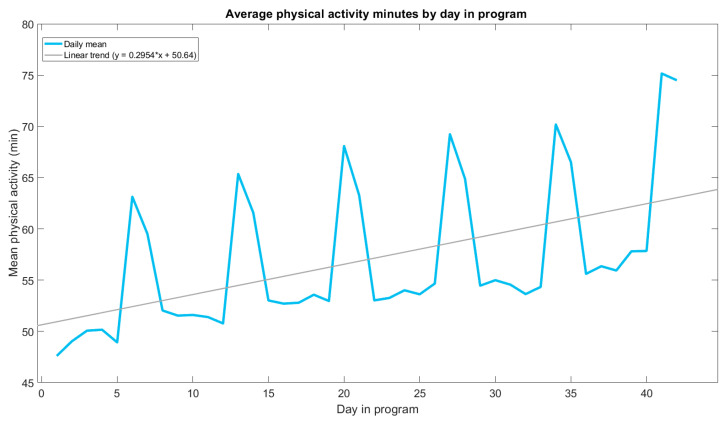
Average physical activity minutes by day in program. Note: includes 337,201 recorded active days across 11,575 participants. Program is 42 days in duration. Day 1 is a Monday.

Table 1 describes the physical activity outcomes of the program. The median daily duration of physical activity was 45 min (IQR = 30–70 min) with participants recording a median of 34 days of activity data across the 42-day program (IQR = 19–41 days, Table 1). Across the duration of the program, mean daily minutes of activity per person increased (see Figure 3). From day 1 to day 42, the average increase in physical activity was 12.1 min per day. Weekends tended to be approximately 10–15 min higher than weekdays.

**Figure 4:**
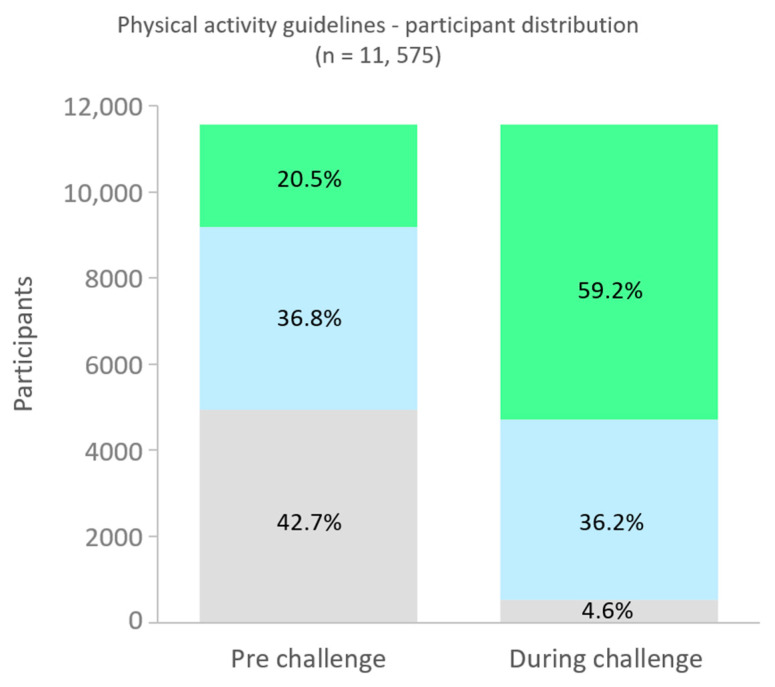
Physical activity guidelines–participant distribution. Note: international physical activity guidelines recommend between 150 and 300 min per week of moderate physical activity.

Of the 11,575 participants who self-reported pre-program physical activity, 42.7% were not meeting international physical activity guidelines, with 36.8% meeting and 20.5% exceeding recommendations (Figure 4). During the study, participants not meeting recommendations reduced significantly to 4.6%, with 36.2% meeting and 59.2% exceeding (χ^2^ (2, N = 11,575) = 5741.81, *p* < 0.001).

### 3.3. Perceived Health Impacts

The multi-level mixed linear regression analysis revealed significant improvements in all five health outcomes measured from baseline to the end of the program (Table 2). Sleep quality showed a 7.6% increase, with the mean score rising from 6.60 to 7.10 out of 10 (*p* < 0.001). Mood also improved by 7.1%, increasing the mean score from 6.94 to 7.43 (*p* < 0.001). Participants reported an 11.6% boost in energy levels, with the mean score climbing from 6.44 to 7.19 (*p* < 0.001). Overall self-rated health saw a 7.7% improvement, going from 6.90 to 7.43 on average (*p* < 0.001). The largest gain was in perceived fitness, which increased by 14.0% from a baseline of 6.11 to 6.97 at the end of the program (*p* < 0.001). All health metric changes were statistically significant based on the multi-level modelling. Individual Cronbach’s alphas for the five health outcome items ranged from 0.8 to 0.86, with an overall α = 0.86, which is above an acceptable level of internal consistency (Table S3).

### 3.4. Satisfaction Survey Items

User feedback was broadly supportive of the program, with low disagreement across all positively framed statements (1–11% disagreement). The highest agreement rate was 92% for statements “I would recommend the exercise challenge to a colleague” and “I would likely participate in the exercise challenge again in future”. The lowest agreement was 42% for the statement “I felt a reduction in levels of stress throughout the challenge” (see Figure S1).

## 4. Discussion

This study set out to evaluate the effectiveness of the 15 Minute Challenge, a workplace wellness initiative, based on real-world user data from 11,575 participants, from 73 Australian, New Zealand, and UK companies. The findings of this study confirmed the hypothesis that the 15 Minute Challenge, a workplace wellness initiative leveraging gamification principles, would lead to substantial increases in physical activity levels and significant improvements in self-reported measures of health. Findings suggested that participants’ physical activity levels increased substantially, with the percentage of participants meeting and exceeding international physical activity increasing substantially. Furthermore, participants reported significant improvements in self-reported measures of health, including fitness, energy, overall health, sleep quality, and mood. Program satisfaction was high, with 92% of participants stating they would recommend the exercise challenge to a colleague and would likely participate again in the future. The program had high engagement throughout its duration, with approximately 98% of users recording data weekly in week 1, declining to approximately 71% of users recording physical activities in the last week of the challenge. The daily duration of physical activity logged in the program significantly increased across the course of the intervention.

The marked improvement in physical activity levels observed in our study aligns with existing literature, emphasizing the efficacy of workplace interventions in promoting physical activity. Our findings revealed a significant uptick in physical activity, with 96% of participants meeting or exceeding physical activity guidelines by the end of the program (a 33% improvement from the baseline level of 63%, Figure 4). Similar workplace interventions, as documented in prior studies, have also demonstrated positive outcomes, albeit with varying degrees of impact. For instance, findings from a previous systematic review of workplace mobile health physical activity interventions found that over half (56%) of included studies reported a significant increase in physical activity, with effective intervention components including self-monitoring, feedback, goal setting, and social comparison [20]. The consistency in physical activity enhancement observed in our study with previous findings underscores the potential of targeted, well-structured interventions like the 15 Minute Challenge in the workplace setting.

In addition to boosting physical activity levels, the 15 Minute Challenge led to notable improvements in various self-reported health measures, including fitness, energy, overall health, sleep quality, and mood. These results are consistent with previous studies that have demonstrated the positive health impacts of workplace physical activity interventions. For instance, research by Stephenson et al. [21] found that an 8-week intervention for full-time desk-based employees consisting of a mobile app led to increased daily breaks and time in short sedentary bouts (<20 min) on weekends compared to the control group, with statistically significant changes in shortest sedentary bouts (5–10 min) on weekends. Similarly, a review by Proper et al. [22] highlighted that workplace interventions could effectively improve physical and mental health outcomes, although the magnitude of these benefits can vary based on the intervention’s design, duration, and intensity. In our study, the most pronounced improvement was observed in participants’ fitness levels, which increased by 14%. This finding underscore the potential of regular, short-duration physical activity to substantially enhance perceived physical fitness [23].

The engagement and satisfaction results from our study indicate high levels of participant commitment and positive reception towards the 15 Minute Challenge, with a notable majority expressing willingness to recommend the program and participate again. Such high engagement rates and positive feedback compare favorably with previous studies on workplace wellness initiatives, where participant satisfaction often varies based on the nature and implementation of the intervention. For example, a previous 8-week pilot study among full-time desk-based employees evaluating two alternative interventions (mobile app n = 20; and mobile app plus sit-stand work desks n = 20) versus controls (n = 16) reported more modest satisfaction (58% app only; 39% app + desk) [21]. It is possible that the favorable comparison may be due to the team-based nature of the 15 Minute Challenge, or its gamified elements. Our study found that engagement throughout the 6-week intervention period ranged between 71% and 98%, which appears consistent with other workplace physical activity programs. For instance, Stephenson et al.’s study [21] reported 59% of participants remained engaged with the app by the end of the intervention, compared to 71% in our study. The engagement rates achieved in our study and other workplace physical interventions appear relatively favorable compared with physical activity interventions in other contexts, which typically struggle with lower engagement levels, underscoring the benefit of delivering interventions in this setting.

This study’s findings provide encouraging evidence highlighting the apparent positive impact of structured workplace wellness programs in increasing physical activity and improving health outcomes. This is particularly important given our increasingly sedentary lifestyles. Such programs may offer benefits to employees through enhanced wellbeing and physical and mental health, with rippling effects potentially leading to increased productivity, reduced absenteeism, and heightened job satisfaction.

Future research is needed to further examine the effectiveness of the 15 Minute Challenge using a more rigorous research design. Ideally, a randomized controlled trial design, employing high-quality, objective, outcome measurement tools, would be conducted to confirm the beneficial results identified in this analysis. Furthermore, studies are needed which go beyond evaluating the immediate impacts of workplace wellbeing initiatives. Such research would examine how to sustain physical activity to influence long-term health outcomes, and examine their impact on downstream outcomes, such as employee morale and organizational productivity. Though engagement with the program was generally quite favorable, a modest downward trend was identified over the course of the program. Therefore, future iterations could experiment with strategies to boost engagement during the program, such as resetting teams or the addition of new incentives, for example, through gamification features such as “streaks” [24].

### Limitations

Before further considering our study’s findings and implications, limitations should be acknowledged. Firstly, the reliance on self-reported measures for physical activity and health outcomes introduces potential biases, including possible overestimation of physical activity levels and subjective interpretation of health improvements. Objective measures, such as wearable fitness trackers, could provide more accurate and reliable data. The absence of a control group limits the ability to attribute observed changes directly to the intervention. The study’s retrospective cohort design may be considered both a strength and a weakness. Demographic data were not collected from participants at baseline to minimize participation burden and reduce barriers to engagement. At the time, the app survey was not designed to capture personal and demographic information, as it was primarily intended as a practical intervention rather than a research project. However, data were gathered under real-world conditions from a large number of users, enhancing the study’s ecological validity and generalizability. The lack of personal and demographic data is a potential limitation, but it also reflects the app’s real-world implementation and usage conditions. Ultimately, the program aimed to deliver an accessible intervention, and collecting data under authentic circumstances from diverse users improved the study’s applicability to actual workplace settings. However, this design cannot establish causality or control for all potential confounding variables that might influence the results. Acknowledging these limitations is crucial when interpreting the study’s results and for informing the design of future research in this area. While the overall findings demonstrate the efficacy of the 15 Minute Challenge in increasing physical activity and improving health metrics, future analyses examining potential moderating factors such as gender, age, country, field of activity, level of education on fitness, energy, general health, mood, and sleep quality could provide valuable insights into optimizing the program’s effectiveness across diverse participant subgroups. Collecting this information in future research studies utilizing this workplace wellness initiative will be essential for understanding and addressing these variables.

## 5. Conclusions

In conclusion, our study provides preliminary evidence that the 15 Minute Challenge, a workplace wellness program, significantly enhances physical activity levels and health outcomes among employees. Key findings indicate substantial increases in participants’ physical activity, with a significant majority meeting or exceeding international guidelines by the program’s end. Participants also reported marked improvements in fitness, energy, overall health, sleep quality, and mood. High program satisfaction and a willingness to participate again suggest the initiative’s positive reception across diverse occupational sectors. Further optimization of program features and controlled, prospective evaluation will help refine this concept into an evidence-based tool for improving population health in the workplace setting.

## Figures and Tables

**Table 1:** Physical activity characteristics.

Physical Activity Outcomes	*n*	Median	IQR
Across 6-week program			
Active participants (≥1 day)	11,575		
Per company		110	63–248
Active days	338,529 (69.6% of total program days)		
Per participant		34	19–41
Active minutes	19,169,511		
Per day		45	30–70
Final week only (week 6)			
Active participants (≥ 1 day)	8189 (70.7%)		
Per company		112 (97)	10–456
Active days	46,734 (82% of total days in final week)		
Per participant		6 (2)	1–7
Active minutes	2,883,270		
Per day		45	30–75

**Table 2:** Results of multi-level mixed linear regression regarding health outcomes (n = 4010 participants).

	Baseline	End of Program	Percent Change	*p*
	Coeff (95% CI)	Coeff (95% CI)		
Sleep	6.60 (6.54–6.67)	7.10 (7.05–7.15)	+7.6%	<0.001
Mood	6.94 (6.87–7.01)	7.43 (7.38–7.49)	+7.1%	<0.001
Energy	6.44 (6.36–6.51)	7.19 (7.14–7.25)	+11.6%	<0.001
Overall health	6.90 (6.85–6.96)	7.43 (7.39–7.48)	+7.7%	<0.001
Fitness	6.11 (6.04–6.17)	6.97 (6.92–7.02)	+14.0%	<0.001

Note: Outcomes scored on a 0–10 scale.

## Data Availability

Access to the data, which are part of an industry collaboration, is confidential and requires contacting the 15 Minute Challenge. Requests for data access will be evaluated on a case-by-case basis, and access will be granted solely to researchers demonstrating a legitimate need for the data and agreeing to maintain its confidentiality.

## Supplementary Materials

The following supporting information can be downloaded at: https://www.mdpi.com/article/10.3390/healthcare12131255/s1, Table S1: List of user feedback survey questions. Table S2: Registered participant characteristics. Table S3: Cronbach’s Alpha Values for Individual Health Outcome Items and Overall Score. Figure S1: Satisfaction survey results.
