# Supplementary material for: Evaluation of the “15 Minute Challenge”: A Workplace Health and Wellbeing Program
Source: Healthcare (Basel). 2024 Jun 24;12(13):1255. doi: 10.3390/healthcare12131255 (PMC11241525; doi:10.3390/healthcare12131255)

**Supplementary content**

**Table S1. List of user feedback survey questions.**

| **Question** | **Strongly disagree** | **Disagree** | **Neutral** | **Agree** | **Strongly agree** |
|---|---|---|---|---|---|
| 1. The exercise challenge motivated me to exercise more than I otherwise would have throughout the 6 weeks | | | | | |
| 2. I intend to make long term changes to my lifestyle due to participating in the exercise challenge | | | | | |
| 3. I believe the challenge has been of benefit to my health and well-being | | | | | |
| 4. I believe participating in the challenge made me more energetic and productive at work | | | | | |
| 5. The challenge had me interacting in a fun way with my colleagues and/or improved team morale | | | | | |
| 6. I felt a reduction in levels of stress throughout the challenge. | | | | | |
| 7. I felt an improvement in mental resilience throughout the challenge. | | | | | |
| 8. I would recommend the exercise challenge to a colleague | | | | | |
| 9. I would likely participate in the exercise challenge again in future | | | | | |
| **Health and wellbeing questions** | | | | | |
| 1. Rate your sleep over the past 7 days on a scale of 0-10 (0 = poor; 10 = excellent) | | | | | |
| 2. Rate your mood over the past 7 days on a scale of 0-10 (0 = poor; 10 = excellent) | | | | | |
| 3. Rate your energy over the past 7 days on a scale of 0-10 (0 = poor; 10 = excellent) | | | | | |
| 4. Rate your overall health over the past 7 days on a scale of 0-10 (0 = poor; 10 = excellent) | | | | | |
| 5. Rate your fitness level over the past 7 days on a scale of 0-10 (0 = poor; 10 = excellent) | | | | | |

**Table S2: Registered participant characteristics.**

| *Participant characteristics* | *n* | Median | IQR |
|---|---|---|---|
| **Companies** | **73** | | |
| **Teams** | **2,105** | **19** | **12 - 46** |
| **Expressions of interest** | **13,116** | **110** | **63 - 248** |
| **Industry** | | | |
| Public Administration and Safety | 15 | | |
| Information Media and Telecommunications | 13 | | |
| Health Care and Social Assistance | 9 | | |
| Education and Training | 7 | | |
| Financial and Insurance Services | 4 | | |
| Manufacturing | 4 | | |
| Other Services | 4 | | |
| Electricity, Gas, Water and Waste Services | 3 | | |
| Public Administration | 3 | | |
| Arts and Recreation Services | 2 | | |
| Professional, Scientific and Technical Services | 2 | | |
| Retail Trade | 2 | | |
| Wholesale Trade | 2 | | |
| Accommodation and Food Services | 1 | | |
| Construction | 1 | | |
| Mining | 1 | | |

**Table S3. Cronbach's Alpha Values for Individual Health Outcome Items and Overall Score**

| Item | Observations | Sign | Item-test correlation | Item-rest correlation | Average Inter-item covariance | alpha |
|---|---|---|---|---|---|---|
| | | | | | | |
| sleep | 8018 | + | 0.7295 | 0.5701 | 1.872734 | 0.8579 |
| mood | 8018 | + | 0.7969 | 0.6761 | 1.758878 | 0.831 |
| energy | 8018 | + | 0.8755 | 0.7898 | 1.564924 | 0.8007 |
| Overall health | 8018 | + | 0.8379 | 0.7427 | 1.706905 | 0.8156 |
| fitness | 8018 | + | 0.7716 | 0.623 | 1.761143 | 0.8457 |
| | | | | | | |
| Test scale | | | | | 1.732917 | 0.8599 |

## Figure S1: Satisfaction survey results

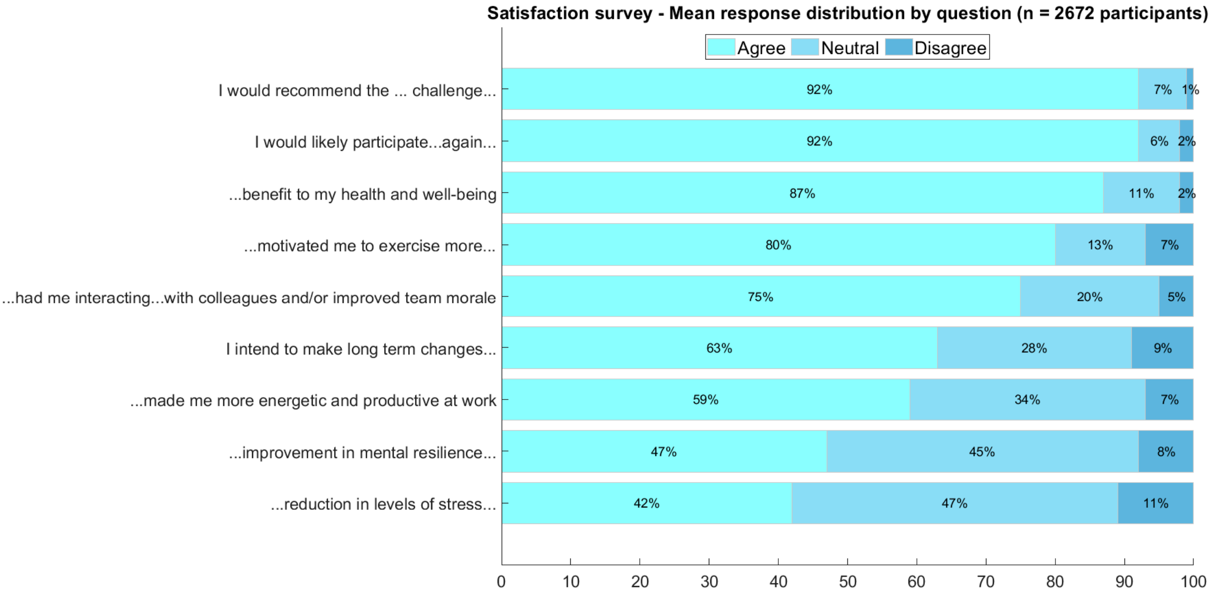

Supplement: Supplementary file 1 [file healthcare-12-01255-s001.zip › healthcare-3016963-supplementary.pdf]
